# A Retrospective Study on Statins and Post-stroke Patients: What About Functional Outcome and Follow-Up in a Stroke Rehabilitation Cohort?

**DOI:** 10.3389/fneur.2021.744732

**Published:** 2021-10-21

**Authors:** Chiara Mele, Giorgio Maggioni, Andrea Giordano, Clara Lunardon, Francesca Balsamo, Alessandra Mazzone, Caterina Pistarini

**Affiliations:** ^1^Department of Clinical-Surgical, Diagnostic and Pediatric Sciences, University of Pavia, Pavia, Italy; ^2^Neurorehabilitation Division, Istituti Clinici Scientifici Maugeri SPA SB, Institute of Veruno, Istituto di Ricovero e Cura a Carattere Scientifico (IRCCS), Novara, Italy; ^3^Bioengineering Division, Istituti Clinici Scientifici Maugeri SPA SB, Institute of Veruno, Istituto di Ricovero e Cura a Carattere Scientifico (IRCCS), Novara, Italy; ^4^Department of Physical and Rehabilitation Medicine, Istituti Clinici Scientifici Maugeri SPA SB, Institute of Genoa Nervi, Istituto di Ricovero e Cura a Carattere Scientifico (IRCCS), Genoa, Italy

**Keywords:** statin, stroke, disability, functional outcome, follow-up

## Abstract

**Objective:** Statins exert pleiotropic effects by influencing several mechanisms, including synaptogenesis, neurogenesis, cerebral flow regulation, and angiogenesis. Results from *in vitro* and animal models suggest that statins could have beneficial effect on functional recovery and outcome after stroke events. However, results in human studies are still controversial. The aim of our study was to evaluate the role of statin in influencing functional outcome and subsequent clinical follow-up in a large cohort of post-stroke rehabilitation patients.

**Methods:** This retrospective study consecutively enrolled 413 adult patients with stroke event, admitted to the division of Neurorehabilitation of the IRCCS ICS Maugeri, Veruno (Italy), for an individual rehabilitation program between 2015 and 2017. Follow-up lasted 3–5 years after discharge. Demographic data, etiology, classification, and anatomical site of stroke lesion, functional assessment, use and duration of statin therapy, and death during hospitalization were collected at baseline and on discharge. Clinical data on subsequent follow-up were also evaluated, considering these as variables: stroke recurrence, bone fractures, cardiovascular complications, and death.

**Results:** In our cohort, 177 patients (42.9%) were prescribed statin therapy, of whom 50 (28.2%) before the stroke event and 127 (71.8%) at the beginning of the rehabilitation process. The use and type of statin therapy as well as the duration of treatment were not associated with recovery and functional outcome, regardless of confounders including sex, age, etiology, and site of stroke lesion, and initial functional level. For what concern post-discharge clinical follow-up, the use of statin therapy was significantly associated with a lower risk of bone fractures (OR = 0.095, CI 95%: 0.012–0.743, *p* = 0.01) independently from age, sex, initial and final functional level, and comorbidities.

**Conclusions:** The use of statins does not seem to influence the functional outcome in post-stroke patients. However, they could exert a protective role against bone fractures during post-discharge follow-up, suggesting further evaluation on this topic.

## Introduction

Statins, also known as HMG-CoA reductase inhibitors, represent a widely used class of cholesterol-lowering medications, able to reduce morbidity and mortality in individuals at high risk of cardiovascular diseases (1). Several randomized clinical trials demonstrated that statin prevents stroke in patients with cardiovascular risk factors and in survivors of first stroke (2, 3).

Stroke events are often associated with short- and long-term disability including immobilization, gait and balance impairment, cognitive deficits, and increased risk of falling and bone fractures (4, 5).

Results from animal models suggested that statins could have beneficial effects on functional and clinical outcomes in post-stroke patients (6–10). Studies on mouse models of acute stroke demonstrated that statin therapy is able to reduce infarct volume and functional disability, enhancing neurological function, synaptogenesis, angiogenesis, and migration of neuronal progenitor cells in the infarct region (6–10). Several pleiotropic properties of statin, including antithrombotic, antioxidant, anti-inflammatory, and neuroprotective effects, probably mediate these benefits (11). However, evidences from clinical studies investigating the effects of statins on post-stroke neurological and functional outcomes are conflicting, and several potential confounders often complicate the interpretation of results (12–16).

Stroke represents also a major risk factor for osteoporosis and bone fractures, which can negatively affect functional recovery, thus increasing disability and mortality risk (5, 17). An interesting beneficial effect of statins on bone metabolism has been documented. The potential association between statins and bone health was described by Mundy et al. (18). The authors observed that statins promote bone formation, through increasing the production of bone morphogenic protein-2 (BMP-2) in mouse bone cells. More recent studies reported that statin could exert anabolic and antiresorptive effects by reducing osteoclast formation and preserving osteoblasts (19–21). Even in this context, results of clinical studies are conflicting (22–27). In fact, several observational studies in humans reported a lower risk of bone fractures in patients treated with statins (22–24), whereas a cohort study in Japanese population observed an inverse association between statin use and bone mineral density (BMD) (26).

To date, it is unclear whether the use of statins in humans can actually improve functional outcome and reduce the risk of bone fractures. Therefore, the aim of our study was to evaluate the role of statins in influencing the functional outcome, the subsequent clinical follow-up, and the risk of fractures in a large cohort of post-stroke rehabilitation patients.

## Materials and Methods

### Study Design and Population

In this observational retrospective study, we included all patients with stroke consecutively admitted to the division of Early Intensive Neurorehabilitation Unit of the IRCCS ICS Maugeri of Veruno, Italy, between January 1, 2015 and December 31, 2017. Collection and analysis of clinical data were performed after approval by the ethics committee of ICS Maugeri and in accordance with the ethical standards laid down in the Declaration of Helsinki. Participants, or authorized representatives, signed a written informed consent before admission to Neurorehabilitation unit.

The inclusion criteria were the following: (1) age ≥ 18 years, (2) diagnosis of stroke on presentation, (3) admission to a hospital emergency department within 24 h of injury, (4) admission within 1 month from the injury to the rehabilitation unit to continue clinical care and rehabilitation program, (5) up to 2 months of observation in the rehabilitation setting, and (6) availability of clinical information on post-discharge follow-up.

Exclusion criteria were pre-existing neurological diseases and/or functional disability and pregnancy.

### Variables, Data Sources, and Measurements

From patients' hospital electronic records, age at occurrence of stroke event, sex, etiology and anatomical site of stroke lesion, comorbidities, functional assessments, use and duration of statin therapy, death during hospitalization were collected at baseline, during the rehabilitation workup and on discharge. Clinical data on post-discharge follow-up were also evaluated.

### Stroke Classifications

Strokes were primarily classified into the two main types: ischemic or hemorrhagic. Stroke type and location were assessed using radiological imaging including computed tomography (CT) and/or magnetic resonance imaging (MRI).

The ischemic stroke subtypes were determined by the Trial of Org 10172 in Acute Stroke Treatment (TOAST) classification (28), which includes five categories: (1) large-artery atherosclerosis, (2) cardioembolism, (3) small-artery occlusion (lacune), (4) stroke of other determined etiology, and (5) stroke of undetermined etiology. Diagnoses are based on clinical features and on data collected by tests such as brain imaging (CT/MRI), cardiac imaging (echocardiography, etc.), duplex imaging of extracranial arteries, arteriography, and laboratory assessments for a prothrombotic state. Ischemic stroke events were also classified into four categories according to the brain territory involved using the Oxfordshire Community Stroke Project (OCSP) (29): (1) total anterior circulation infarct (TACI), (2) partial anterior circulation infarct (PACI), (3) posterior circulation infarct (POCI), or lacunar infarct (LACI) based on their maximum neurological defects.

Hemorrhagic strokes were classified according to the type and location of bleeding: (1) typical intracerebral hemorrhage, (2) atypical intracerebral hemorrhage, (3) subarachnoid hemorrhage (SAH).

### Comorbidities

Comorbidity at the time of admission to our neurorehabilitation unit was assessed with the Cumulative Illness Rating Scale Geriatric Version (CIRS-G) (30). CIRS-G is a valid instrument in younger and elderly patients (31). The score differentiates among 14 organ systems. Every comorbidity of a patient was assigned to one of the organ systems and rated from 1 (mild comorbidity) to 4 (extremely severe comorbidity).

### Rehabilitation Outcome and Follow-Up

Rehabilitation outcomes were evaluated through the Functional Independence Measure (FIM) scale, an 18-item measurement tool that explores the physical, psychological, and social function of an individual (32, 33). The tool is used to assess the patient's level of disability as well as change in patient status in response to rehabilitation or medical intervention (34). FIM scale was evaluated on admission and at discharge.

All patients were followed up from 3 to 5 years after discharge. Data about the occurrence of bone fractures, stroke recurrence, cardiovascular (CV) complications, and death were collected.

### Statistical Analysis

Values are expressed as median and interquartile range (IQR) or absolute number and percentage. Data were tested for normality of distribution with the Shapiro–Wilk test and log-transformed when needed in order to correct for skewness. Mann–Whitney and chi-square tests were used for comparisons between groups. Multiple linear regression analysis was used to evaluate the predictive role of the use and duration of statin therapy on functional outcome, adjusted for age, sex, etiology and site of stroke lesion, initial functional level and comorbidities. A multinomial regression model was also used to evaluate the association between the post-discharge bone fractures occurrence and the use of statin therapy. A value of *p* < 0.05 was considered as statistically significant. All statistical analyses were performed using SPSS Statistics 21 (IBM Corporation, Somers, NY, USA).

## Results

A total of 413 adult patients with stroke event were consecutively admitted to our Neurorehabilitation Unit for an individual rehabilitation program from January 2015 to December 2017.

Clinical characteristics, functional outcome, and follow-up data of the whole population are summarized in Table 1. Most of patients (85.5%) were over 65 years of age at the time of stroke event. Male sex was more prevalent than female (54.5 vs. 45.5%).

**Table 1:** Clinical, rehabilitation, and follow-up characteristics of the population as a whole and subdivided into two groups according to the use or not of statin therapy.

**Variables**		**Whole population** **(*n* = 413)**	**Statin** **(*n* = 177, 42.9%)**	**No statin** **(*n* = 236, 57.1%)**	***p*-value**
**Age** (years), *median* (*IQR*)		78 (69–84)	78 (71–83)	79 (69–84)	0.27
**Sex**	M	225 (54.5%)	100 (56.5%)	125 (53.0%)	0.47
	F	188 (45.5%)	77 (43.5%)	111 (47.0%)	
**Statin—active substance**	Simvastatin	–	20 (11.3%)	–	–
	Atorvastatin	–	152 (85.9%)	–	–
	Rosuvastatin	–	5 (2.8%)	–	–
**Type of lesion**	**Ischemic**	341 (82.6%)	**162 (91.5%)**	**179 (75.8%)**	**<0.0001**
	**Haemorrhagic**	72 (17.4%)	**15 (8.5%)**	**57 (24.2%)**	
**TOAST** (*for ischaemic stroke only*)	Large-artery atherosclerosis	91 (26.7%)	48 (29.6%)	42 (23.5%)	0.20
	Cardioembolism	97 (28.4%)	40 (24.7%)	56 (31.3%)	0.18
	Small-artery occlusion	115 (33.7%)	58 (35.8%)	53 (29.6%)	0.22
	Other etiology	34 (10.0%)	16 (9.9%)	24 (13.4%)	0.31
	Undetermined	4 (1.2%)	0 (0.0%)	4 (2.2%)	0.06
**OCSP** (*for ischaemic stroke only*)	TACI	86 (25.2%)	36 (22.2%)	50 (27.9%)	0.23
	PACI	49 (14.4%)	29 (17.9%)	20 (11.2%)	0.09
	POCI	57 (16.7%)	24 (14.8%)	33 (18.4%)	0.37
	LACI	149 (43.7%)	73 (45.1%)	76 (42.5%)	0.63
**Haemorrhagic stroke etiology**	Typical ICH	24 (33.3%)	5 (33.3%)	19 (33.3%)	1.00
	Atypical ICH	37 (51.4%)	8 (53.3%)	29 (50.9%)	0.86
	SAH	11 (15.3%)	2 (13.4%)	9 (15.8%)	0.81
**Location**	Frontal lobe	22 (5.3%)	8 (4.5%)	14 (5.9%)	0.53
	Parietal lobe	33 (8.0%)	16 (9.0%)	17 (7.2%)	0.49
	Temporal lobe	11 (2.7%)	1 (0.6%)	10 (4.2%)	0.06
	Occipital lobe	11 (2.7%)	4 (2.3%)	7 (3.0%)	0.66
	Cerebellum	19 (4.6%)	7 (4.0%)	12 (5.1%)	0.59
	Basal Ganglia	130 (31.4%)	62 (35.0%)	68 (28.8%)	0.18
	Brain stem	17 (4.1%)	9 (5.1%)	8 (3.4%)	0.39
	Multiple	170 (41.2%)	70 (39.5%)	100 (42.4%)	0.56
**CIRS-G**, *median* (*IQR*)	Total score	18 (13–22)	17 (14–21)	18 (13–22)	0.73
	Severity index	1.3 (0.9–1.6)	1.3 (1.0–1.6)	1.3 (0.9–1.6)	0.42
**FIM**, *median* (*IQR*)	Total score T0	52 (33–74)	52 (28–73)	53 (37–75)	0.24
	Total score T1	82 (51–106)	82 (52–105)	82 (51–107)	0.69
	Δ total score	19 (8–35)	20 (10–34)	18 (7–35)	0.24
	Motor score T0	25 (16–43)	24 (14–43)	25 (17–44)	0.35
	Motor score T1	55 (30–74)	52 (29–73)	55 (32–75)	0.86
	Δ motor score	16 (6–30)	17 (8–31)	16 (6–30)	0.34
	Cognitive score T0	27 (16–33)	27 (15–33)	27 (18–33)	0.49
	Cognitive score T1	30 (22–34)	30 (21–34)	30 (22–34)	0.78
	Δ cognitive score	1 (0–4)	1 (0–4)	1 (0–3)	0.19
**Death during hospitalization**		31 (7.5%)	14 (7.9%)	17 (7.2%)	0.79
**Post-discharge** **follow-up**	Stroke recurrence	42 (10.2%)	16 (9.0%)	26 (11.0%)	0.51
	**Bone fractures**	16 (3.9%)	**1 (0.6%)**	**15 (6.4%)**	**0.002**
	CV complications	13 (3.1%)	4 (2.3%)	9 (3.8%)	0.37
	Death	2 (0.5%)	0 (0.0%)	2 (0.8%)	0.22

*Data are expressed as median and interquartile range (IQR) or absolute number and percentage. Comparison between groups were performed with χ^2^ or Mann–Whitney tests*.

*TACI, total anterior circulation infarct; PACI, partial anterior circulation infarct; POCI, posterior circulation infarct; LACI, lacunar infarct; ICH, intracerebral hemorrhage; T0 on admission to neurorehabilitation, T1 at discharge; CV, cardiovascular; TOAST, Trial of Org 10172 in Acute Stroke Treatment; OCSP, Oxfordshire Community Stroke Project; SAH, subarachnoid hemorrhage; CIRS-G, Cumulative Illness Rating Scale-Geriatric; FIM, Functional Independence Measure scale*.

*Significant differences are shown in bold characters*.

Overall, an ischemic lesion was detected in 341 patients (82.6%) whereas a hemorrhagic lesion in 72 patients (17.4%). As regard the localization of the stroke lesion, most patients (41.2%) presented multiple site lesions, with basal ganglia (31.4%) being the most involved. According to the TOAST classification, the most prevalent ischemic stroke etiology was the small-artery occlusion (33.7%), followed by cardioembolism and large-artery atherosclerosis (28.4 and 26.7%, respectively). According to OCSP classification, the brain territory most frequently involved in ischemic lesions was LACI (43.7%). In case of hemorrhagic lesions, atypical intracerebral hemorrhages were the most represented (51.4%).

Approximately half of the patients (42.9%) were prescribed statin therapy, of whom 50 (28.2%) before the stroke event and 127 (71.8%) at the beginning of the rehabilitation process. The most prescribed statin was atorvastatin (85.9%), followed by simvastatin (11.3%), and rosuvastatin (2.8%). There were no severe drug-related toxic effects during hospitalization and statin therapy was continued for the entire course of the hospital stay in the rehabilitation unit and after discharge.

Death during rehabilitative hospitalization was observed in 31 patients (7.5%).

For what concern post-discharge follow-up, stroke recurrence was observed in 42 patients (10.2%), bone fracture in 16 patients (3.9%), cardiovascular complications in 13 patients (3.1%) and death in two cases.

Comparison analyses were conducted between patients with and without statin therapy (Table 1). As expected, statin therapy was more frequently prescribed in ischemic stroke than in hemorrhagic events (χ^2^ = 17.3, *p* < 0.0001). No significant differences were found between the two groups in terms of demographic, clinical, and functional outcomes, whereas patients using statin therapy had a significant lower prevalence of bone fractures during post-discharge follow-up compared with those who were not using statin therapy (χ^2^ = 9.1, *p* = 0.002).

By analyzing functional outcome and post-discharge follow-up in patients treated with statin subgrouped according to the beginning of treatment, no significant differences were found in terms of FIM and post-discharge complications between patients who started statin therapy before stroke event and patients who started treatment at the beginning of rehabilitation process (Table 2).

**Table 2:** Rehabilitative and follow-up characteristics of patients treated with statin subdivided into two groups according to the beginning of treatment: patients who started statin therapy before stroke even and patients who started treatment at the beginning of rehabilitation process.

**Variables**		**Statin** **before stroke** **(*n* = 50)**	**Statin during** **rehabilitation** **(*n* = 127)**	***p*-value**
**CIRS-G**, *median* (*IQR*)	Total score	19 (15–22)	17 (13–20)	0.11
	Severity index	1.4 (1.1–1.6)	1.3 (1.0–1.6)	0.87
**FIM**, *median* (*IQR*)	Total score T0	53 (32–72)	52 (28–73)	0.99
	Total score T1	78 (60–103)	83 (52–107)	0.95
	Δ total score	19 (8–38)	21 (11–34)	0.79
	Motor score T0	24 (15–43)	24 (14–43)	0.82
	Motor score T1	49 (33–70)	54 (29–75)	0.78
	Δ motor score	17 (8–30)	17 (8–31)	0.81
	Cognitive score T0	29 (19–33)	27 (13–32)	0.59
	Cognitive score T1	30 (24–33)	30 (20–34)	0.40
	Δ cognitive score	1 (0–3)	1 (0–4)	0.87
**Death during hospitalization**		4 (8.0%)	10 (7.9%)	0.91
**Post-discharge** **follow-up**	Stroke recurrence	2 (4.0%)	14 (11.0%)	0.26
	Bone fractures	0 (0.0%)	1 (0.8%)	0.11
	CV complications	1 (2.0%)	3 (2.4%)	0.88
	Death	0 (0.0%)	0 (0.0%)	–

*Data are expressed as median and interquartile range (IQR) or absolute number and percentage. Comparison between groups were performed with χ^2^ or Mann–Whitney tests*.

*T0 on admission to neurorehabilitation, T1 at discharge*.

### Associative Analyses

Multiple linear regression analysis was conducted to evaluate the predictive role of the use of statin therapy on functional outcome. The use and type of statin therapy were not associated with the recovery and functional outcome in terms of FIM total score T1, regardless of confounders including age, sex, etiology and site of stroke lesion, initial functional level expressed as basal FIM and comorbidities (CIRS-G) (Supplementary Table 1). A secondary analysis was performed in the subgroup of statin users to evaluate the association between the duration of statin treatment and functional outcome. Also, the duration of treatment did not represent a predictor of recovery and functional outcome, independently from the potential confounding variables mentioned above (Supplementary Table 2). Basal FIM (FIM total score T0) emerged as the only independent predictor of recovery and functional outcome among the included variables.

For what concern post-discharge clinical follow-up, the use of statin therapy, in particular atorvastatin, was significantly associated with a lower risk of bone fractures (OR = 0.095, CI 95%: 0.012–0.743, and *p* = 0.01) independently from age, sex, initial and final functional level, and comorbidities (Table 3).

**Table 3:** Multinomial regression model evaluating the negative association between statin therapy and bone fractures.

**Covariates**		**Bone fractures**		
		**OR**	**CI 95%**	***p*-value**
**Age**		1.014	0.963–1.068	0.59
**Sex**	M	0.785	0.347–4.064	0.78
	F	1	–	Reference
**CIRS-G total score**		0.929	0.836–1.033	0.18
**FIM total score T0**		1.004	0.962–1.048	0.86
**FIM total score T1**		1.018	0.988–1.050	0.24
**Statin therapy**	Yes	**0.095**	**0.012–0.743**	**0.01**
	No	1	–	Reference

*Dependent variable: bone fractures (No = 0, Yes = 1), covariates: age, sex, CIRS-G total score, FIM total score T0 and T1, statin therapy (No = 0, Yes = 1)*.

*TACI, total anterior circulation infarct; PACI, partial anterior circulation infarct; POCI, posterior circulation infarct; LACI, lacunar infarct; ICH, Intracerebral hemorrhage; T0 on admission to neurorehabilitation, T1 at discharge*.

*Significant differences are shown in bold characters*.

## Discussion

The results of our study show that the use and type of statin therapy as well as the duration of treatment were not significantly associated with the recovery and functional outcome in terms of FIM, regardless of confounders including sex, age, etiology, and site of stroke lesion, initial functional level, and comorbidities. However, during post-discharge clinical follow-up, statin users were found to be at lower risk of developing bone fractures.

Despite currently available treatment options for stroke events, patients often face the prospect of substantial post-stroke disability that could influence the occurrence of other clinical complications and impact quality of life (35). Randomized controlled trials demonstrated that statins are able to prevent ischemic stroke in high-risk patients and in survivors of first stroke (2, 3). More recently, some evidences suggest that statin therapy could improve rehabilitation and functional outcomes after both ischemic and hemorrhagic stroke (12, 15, 16). Animal stroke models showed that statins have microvascular and neuroprotective properties. In mice, pre-treatment with high-dose statin enhances an upregulation of endothelial nitric oxide synthase (eNOS), thus promoting cerebral blood flow and a reduction of infarct volume, and improving neurological function (7, 36). In mice with embolic stroke, low atorvastatin doses are able to induce the expression of vascular endothelial growth factor (VEGF), thus promoting neovascularization, and improve neurological function by enhancing synaptogenesis and proliferation of neural progenitor cells (10). Neuroprotective effects of statins, including antioxidative, anti-inflammatory, angiogenic, neurogenic, and antiapoptotic properties, have also been demonstrated in animals with hemorrhagic stroke (37). All these findings led to hypothesized that statin administration may positively influence cerebral repair after injury acting on neurogenesis and angiogenesis (7, 9, 10, 16, 36).

However, evidences from clinical studies evaluating the effects of statins on stroke rehabilitation and functional outcome are conflicting, and the interpretation of results is often difficult because of small sample size, possible bias, and confounders (12–16). Whereas several observational studies observed a decreased mortality rate or improved functional outcomes in patients treated with statins, these beneficial effects were not demonstrated in other studies (13–16, 38–40). Also, results from randomized controlled trials and meta-analysis were not univocal (12, 41–43). With the aim of clarifying this issue, we evaluated the possible role of statins in influencing the functional outcome in terms of FIM in a large cohort of stroke patients with different etiology, by using a multinomial logistic regression analysis in order to eliminate the effect of possible confounders including age, sex, etiology and site of stroke lesion, initial functional level, and comorbidities.

It is important to point out that most of previous studies used modified Rankin scale (mRS) to evaluate functional outcome. The mRS is a single-item scale for assessing levels of functional independence among stroke survivors. It is used to categorize level of functional independence with reference to pre-stroke activities rather than on observed performance of a specific task (44). Our study evaluated functional outcome using FIM that represents a widely accepted functional assessment measure applied during inpatient rehabilitation (44, 45). The FIM is an 18-item ordinal scale, used with all diagnoses within a rehabilitation population and measures independent performance in self-care, sphincter control, transfers, locomotion, communication, and social cognition (45). With respect to mRS, FIM addresses a greater number of items and an accurate evaluation of several aspects both in motor and cognitive functions.

In contrast to our expectation that statin treatment has a significant beneficial effect on functional outcome after stroke, our results show that the use and type of statin therapy as well as the duration of treatment did not significantly influence the recovery and functional outcome, in agreement with other previous clinical studies (38, 40, 42). Despite the well-known pleiotropic effects of statins and its potential role in influencing rehabilitation and functional outcome in animal models, in clinical practice patients generally have many potential individual and clinical factors that could influence rehabilitation, thus masking the effect of statins. Moreover, most of clinical studies, including ours, evaluated only a short-term functional outcome. Therefore, further clinical trials and experimental model data will be required to elucidate the potential role and effect of statin treatment on short-term and long-term functional outcome.

Our study also evaluated post-discharge clinical follow-up lasted 3–5 years after discharge in terms of stroke recurrence, bone fractures, CV complications, and death. Our results suggest that statin users were at lower risk of developing bone fractures. Many evidences suggest that stroke represent a major risk factor for bone fracture and osteoporosis, as it induces immobilization, balance impairment, gait disability, and increases fall risk (5, 17). Fractures can further impair functional recovery, increasing disability, and mortality risk in these patients (4, 46). Several studies hypothesized a further pleiotropic effect of statins in reducing osteoporosis and bone fractures, but controversial results have been reported (22–27). The potential source of the conflicting results could be related to many factors including ethnicity, the individual predisposition to develop osteoporosis, as well as dosage and duration of statin therapy. A meta-analysis of Jin et al. (47) that included studies on general population showed that statin therapy is significantly associated with a decreased risk of overall fractures. Recently, Lin et al. (24), in a retrospective study specifically focused on stroke patients, reported that statin therapy is able to decrease the risk of osteoporosis and bone fractures of about 30% with a dose effect-relationship. However, these studies did not consider comorbidities as well as the initial and final functional level, which could represent potential confounders. Our results show that the use of statin therapy, and in particular atorvastatin, was significantly associated with a lower risk of bone fractures in post-stroke patients, independently from age, sex, initial and final functional level, and comorbidities, suggesting a potential intrinsic effect of the statin molecule on bone metabolism. The mechanisms underlying the relationship between statins, osteoporosis, and bone fractures have not yet been elucidated. *In vitro* and *in vivo* studies demonstrated that statins increase expression of BMP-2, which has a key role in the processes of bone formation, promote osteogenic activity, and simultaneously inhibit osteoblast activity through different molecular pathways (19–21).

Our study has several limitations, which should be pointed out. First, the retrospective nature of the study does not allow us to draw any conclusion about the mechanisms involved in the negative association between statin and bone metabolism. Second, we did not assess all the individual risk factors that could influence the lack of association between statin use and functional outcome.

In conclusion, in our cohort of post-stroke patients, the use of statins does not seem to influence the functional outcome. However, they could exert a protective role against bone fractures during post-discharge follow-up, suggesting further evaluation on this topic.

## Data Availability Statement

The original contributions presented in the study are included in the article/Supplementary Material, further inquiries can be directed to the corresponding author/s.

## Ethics Statement

The studies involving human participants were reviewed and approved by Istituti Clinici Scientifici Salvatore Maugeri, IRCCS, Veruno. The patients/participants, or their authorized representatives, provided a written informed consent to participate in this study.

## Author Contributions

CM designed the study and drafted the manuscript. GM and AG interpreted the results and contributed to the discussion. CL and FB collected and analyzed data. AM contributed to analyse data and reviewed the manuscript. CP reviewed and edited the manuscript. All authors contributed to the article and approved the submitted version.

## Conflict of Interest

The authors declare that the research was conducted in the absence of any commercial or financial relationships that could be construed as a potential conflict of interest.

## Publisher's Note

All claims expressed in this article are solely those of the authors and do not necessarily represent those of their affiliated organizations, or those of the publisher, the editors and the reviewers. Any product that may be evaluated in this article, or claim that may be made by its manufacturer, is not guaranteed or endorsed by the publisher.
