# Supplementary material for: A Retrospective Study on Statins and Post-stroke Patients: What About Functional Outcome and Follow-Up in a Stroke Rehabilitation Cohort?
Source: Front Neurol. 2021 Oct 21;12:744732. doi: 10.3389/fneur.2021.744732 (PMC8567028; doi:10.3389/fneur.2021.744732)
Supplement: Supplementary file 1 [file Table_1.DOCX]

**Supplementary Table 1.** Multiple linear regression analysis to evaluate the potential association between statin use and functional outcome in the population as a whole. Dependent variable: FIM total score T1/Δ FIM total score, covariates: statin use, type of statin therapy, age, sex, aetiology of stroke lesion, site of stroke lesion, FIM total score T0, CIRS-G.

| **Covariates** | | **FIM total score T1** | | **Δ FIM total score** | |
| --- | --- | --- | --- | --- | --- |
|  |  | **β** | **p-value** | **β** | **p-value** |
| **Statin therapy (No=0, Yes=1)** | | 0.008 | 0.25 | 0.003 | 0.96 |
| **Type of statin therapy**  **(for each item No=0, Yes=1)** | Simvastatin | 0.41 | 0.20 | 0.065 | 0.24 |
|  | Atorvastatin | -0.065 | 0.21 | 0.020 | 0.70 |
|  | Rosuvastatin | 0.026 | 0.40 | 0.044 | 0.43 |
| **Age (years)** | | 0.017 | 0.62 | 0.011 | 0.84 |
| **Sex (M=0, F=1)** | | 0.015 | 0.64 | 0.028 | 0.61 |
| **Aetiology of stroke lesion**  **(Ischemic=0, Haemorrhagic=1)** | | 0.006 | 0.85 | 0.024 | 0.67 |
| **Site of stroke lesion**  **(for each item No=0, Yes=1)** | Frontal lobe | -0.029 | 0.46 | -0.069 | 0.32 |
|  | Parietal lobe | 0.016 | 0.74 | 0.028 | 0.74 |
|  | Temporal lobe | -0.071 | 0.15 | -0.132 | 0.13 |
|  | Occipital lobe | -0.043 | 0.25 | -0.076 | 0.25 |
|  | Cerebellum | -0.019 | 0.61 | -0.031 | 0.63 |
|  | Basal Ganglia | -0.034 | 0.47 | -0.048 | 0.56 |
|  | Brain stem | -0.046 | 0.22 | -0.084 | 0.20 |
|  | Multiple | 0.028 | 0.65 | 0.056 | 0.60 |
| **FIM total score T0** | | 0.82 | <0.0001 | - | - |
| **CIRS-G** | | -0.012 | 0.71 | -0.013 | 0.81 |

*For abbreviation: T0 on admission to neurorehabilitation, T1 at discharge.*
