# Supplementary material for: A Retrospective Study on Statins and Post-stroke Patients: What About Functional Outcome and Follow-Up in a Stroke Rehabilitation Cohort?
Source: Front Neurol. 2021 Oct 21;12:744732. doi: 10.3389/fneur.2021.744732 (PMC8567028; doi:10.3389/fneur.2021.744732)
Supplement: Supplementary file 2 [file Table_2.DOCX]

**Supplementary Table 2**. Multiple linear regression analysis to evaluate the potential association between the duration of statin treatment and functional outcome in the subgroup of statin users. Dependent variable: FIM total score T1/Δ FIM total score, covariates: duration of statin treatment, age, sex, aetiology of stroke lesion, site of stroke lesion, FIM total score T0, CIRS-G.

| **Covariates** | | **FIM total score T1** | | **Δ FIM total score** | |
| --- | --- | --- | --- | --- | --- |
|  |  | **β** | **p-value** | **β** | **p-value** |
| **Duration of statin treatment** | | -0.006 | 0.89 | 0.001 | 0.98 |
| **Age (years)** | | 0.017 | 0.71 | 0.011 | 0.89 |
| **Sex (M=0, F=1)** | | -0.031 | 0.49 | -0.058 | 0.47 |
| **Aetiology of stroke lesion**  **(Ischemic=0, Haemorrhagic=1)** | | -0.007 | 0.88 | -0.008 | 0.93 |
| **Site of stroke lesion**  **(for each item No=0, Yes=1)** | Frontal lobe | 0.019 | 0.75 | 0.038 | 0.73 |
|  | Parietal lobe | 0.041 | 0.59 | 0.072 | 0.61 |
|  | Temporal lobe | -0.104 | 0.21 | -0.183 | 0.23 |
|  | Occipital lobe | -0.010 | 0.90 | -0.118 | 0.13 |
|  | Cerebellum | -0.034 | 0.51 | -0.062 | 0.52 |
|  | Basal Ganglia | 0.029 | 0.70 | 0.062 | 0.66 |
|  | Brain stem | -0.038 | 0.50 | -0.067 | 0.52 |
|  | Multiple | 0.160 | 0.12 | 0.279 | 0.15 |
| **FIM total score T0** | | 0.857 | <0.0001 | - | - |
| **CIRS-G** | | 0.017 | 0.71 | 0.031 | 0.71 |

*For abbreviation: T0 on admission to neurorehabilitation, T1 at discharge.*
